# Development and validation of a preoperative magnetic resonance imaging-based and machine learning model for the noninvasive differentiation of intracranial glioblastoma, primary central nervous system lymphoma and brain metastases: a retrospective analysis

**DOI:** 10.3389/fonc.2025.1541350

**Published:** 2025-04-22

**Authors:** Yuxiang Sun, Junpeng Xu, Dongsheng Kong, Yu Zhang, Qijia Wu, Liqin Wei, Zihao Zhu, Chunhui Li, Shiyu Feng

**Affiliations:** ^1^ Department of Neurosurgery, Affiliated Hospital of Hebei University, Baoding, China; ^2^ Department of Neurosurgery, the First Medical Center of Chinese PLA General Hospital, Beijing, China; ^3^ Department of Neurosurgery, Xuanwu Hospital, Xiongan, China

**Keywords:** central nervous system malignant tumors, machine learning, magnetic resonance imaging; multi-classification, glioblastoma, PCNSL = primary CNS lymphoma

## Abstract

**Background:**

Accurate preoperative identification of intracranial glioblastoma (GB), primary central nervous system lymphoma (PCNSL), and brain metastases (BM) is crucial for determining the appropriate treatment strategy.

**Purpose:**

We aimed to develop and validate the utility of preoperative magnetic resonance imaging-based radiomics and machine learning models for the noninvasive identification them. STUDY TYPE: Retrospective. POPULATION: We included 202 patients, including 71 GB, 59 PCNSL, and 72 BM, randomly divided into a training cohort (n =141) and a validation cohort (n = 61).FIELD STRENGTH/SEQUENCE: Axial T2-weighted fast spin-echo sequence (T2WI) and contrast-enhanced T1-weighted spin-echo sequence (CE-T1WI) using 1.5-T and 3.0-T scanners. ASSESSMENT: We extracted radiomics features from the T2 sequence and CE-T1 sequence separately. Then, we applied the F-test and recursive feature elimination (RFE) to reduce the dimensionality for both individual sequences and the combined sequence CE-T1 combined with T2.The support vector machine (SVM), k-nearest neighbor (KNN), and naive Bayes classifier (NBC) were used in model development. STATISTICAL TESTS: Chi-square test, one-way analysis of variance, and Kruskal-Wallis test were performed. The P values <0.05 were considered statistically significant. Performance was evaluated using AUC, sensitivity, specificity, and accuracy metrics.

**Result:**

The SVM model exhibited superior diagnostic performance with macro-average AUC values of 0.91 for CE-T1 alone, 0.86 for T2 alone, and 0.93 for combined CE-T1 and T2 sequences. And the combined sequence model demonstrated the best overall accuracy, sensitivity, and F1 score, with an accuracy of 0.77, outperforming both KNN and NBC models.

**Conclusion:**

The SVM-based MRI radiomics model effectively distinguishes between GB, PCNSL, and BM. Combining CE-T1 and T2 sequences significantly enhances classification performance, providing a robust, noninvasive diagnostic tool that could assist in treatment planning and improve patient outcomes.

## 1 Introduction

Central nervous system malignant tumors are a type of highly heterogeneous intracranial solid tumors, mainly originating from brain tissue or secondary to tumor metastasis in other organs. Among them, glioblastoma (GB), Primary Central Nervous System Lymphoma,(PCNSL) and brain metastases (BM) are three common CNS malignant tumors (1). Due to their complexity in clinical manifestations and treatment strategies, they cause a serious threat to patients’ health. GB is the most common and primary brain tumor in adults, with a median overall survival of 14-17 months and a 5-year survival rate of less than 5% (2). PCNSL has the worst prognosis among all non-Hodgkin lymphomas, with a 5-year estimated overall survival rate of only 30.5% (3). Although the prognosis of BM has improved, the median survival time of patients is more than 6 months, ranging from 8 to 16 months, but it still depends on the type of primary tumor (4). There are significant differences in treatment options for the three tumors mentioned above—GB, BM, and PCNSL. GB usually adopts maximum surgical resection combined with concurrent chemoradiotherapy (5); BM is mainly treated with surgical resection, possibly supplemented by chemoradiotherapy (6); while PCNSL is mainly treated with chemotherapy (7). Therefore, accurate preoperative identification of intracranial GB, PCNSL, and BM is crucial for formulating a personalized treatment plan (3).Conventional magnetic resonance imaging (cMRI) technology is currently an important tool for preoperative diagnosis and evaluation of brain tumors. However, due to the heterogeneity of tumors and the overlap of imaging features, For example, PCNSL, GB, and BM can all present with homogeneous enhancement accompanied by edema (8, 9), leading to diagnostic delays and suboptimal treatment, There are obvious limitations in identification based solely on neuroradiologists’ experience. Although diffusion-weighted imaging (DWI) and dynamic susceptibility contrast perfusion-weighted imaging (DSC-PWI) are key sequences for differentiating brain tumors. For example, DWI measures water molecule movement, where PCNSL typically exhibits restricted ADC, while GBM and BM show more variable signals. DSC-PWI assesses tumor hemodynamics, with studies showing that PCNSL has significantly lower rCBV than GBM and BM, aiding in clinical differentiation (10). However, these techniques still have limitations, including signal overlap, blood flow effects, and imaging parameter standardization issues (9). Multiple reports (11, 12) pointed out that central nervous system tumors, especially PCNSL, have a very high misdiagnosis rate, which to a certain extent limits the formulation and implementation of individualized treatment strategies. Traditional diagnostic methods that rely on surgeon experience and cMRI still face great challenges in accurately identifying central nervous system malignancies. Although needle biopsy can confirm the type of tumor through pathological analysis, thus making up for the shortcomings of the above identification methods to a certain extent, because it is an invasive operation, it will be accompanied by certain surgical risks, such as postoperative bleeding, infection, and Possible neurological dysfunction (13), which increases the financial and psychological burden on patients. Especially for elderly patients, due to the higher risks of surgery, they are often unable to tolerate the trauma of multiple surgeries.

In order to solve the problem of insufficient specificity of cMRI in the diagnosis of brain tumors and reduce the unnecessary damage and burden of puncture biopsy on patients, the scientific research community has actively explored and implemented many novel imaging technologies in the past few decades. These innovations not only enriched the diagnostic methods, but also introduced many additional imaging parameters, greatly increasing the complexity and depth of neuroimaging data. However, faced with this massive and complex diagnostic information, how to efficiently and economically evaluate its value in clinical practice has become a problem that needs to be solved urgently. More and more studies have shown that preoperative MRI is feasible in differentiating the types of central nervous system tumors (14), and that MRI imaging features have great development value in differentiating tumor types. Artificial intelligence (AI) especially machine learning (ML) technologies, has opened up new auxiliary diagnosis pathways for clinicians. AI-driven imaging data analysis is particularly eye-catching, as it can build models, comprehensively evaluate various types of imaging data, and effectively process massive amounts of data. This advantage not only improves data processing capabilities, but also potentially enhances the comparability and objectivity of diagnostic results, as it no longer relies entirely on the personal experience of clinicians. AI technology has shown great potential and broad application prospects in improving the specificity, efficiency, and accuracy of brain tumor diagnosis. It not only solves many challenges faced by traditional imaging technologies, but also provides more comprehensive, objective, and in-depth data support for clinical decision-making. However, many studies are currently limited to differentiating between two types of central nervous system tumors, such as GB and PCNSL (15–17) or GB and BM (18, 19), but relatively few studies have been conducted on the simultaneous identification of three brain tumors. This study developed three machine learning models based on preoperative MRI. By analyzing the radiomics features of MRI images, we aimed to establish a multi-classification model that can provide high-accuracy differentiation of intracranial GB, PCNSL, and BM to assist clinical decision-making and treatment planning.

## 2 Materials and methods

### 2.1 IRB approval

This study has been approved by the Ethics Committee and publicly registered ([S2018-268-02], approval date: [2018.12]). This study was conducted in strict accordance with the guidelines of the Declaration of Helsinki. All patients included in the study signed written informed consent before enrollment.

### 2.2 Patients

**Figure 1:**
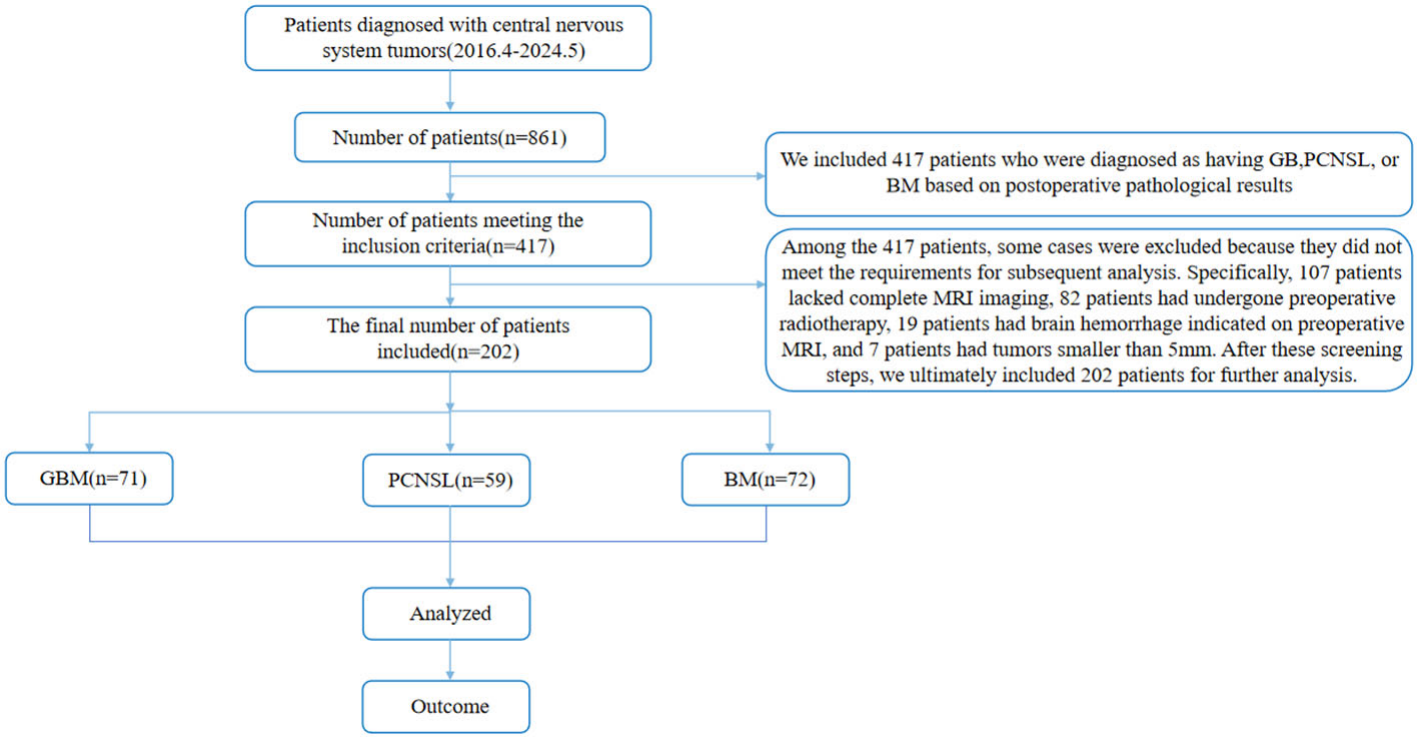
Patient inclusion and exclusion criteria flowchart.

We retrospectively enrolled 202 patients with intracranial space-occupying lesions who underwent surgical treatment and were pathologically confirmed from April 2016 to May 2024. The average age of the patients was 58.7 ± 15.4 years, including 98 male patients and 104 female patients. The specific tumor type distribution was: 71 patients with GB, 59 patients with PCNSL, and 72 patients with BM. Inclusion criteria: ① According to the NCCN Clinical Practice Guidelines for Central Nervous System Tumors issued in 2023, the pathological or molecular diagnosis results were GB, PCNSL, and BM; ② The patients did not receive chemoradiotherapy and surgical treatment before MRI examination; ③ GB, PCNSL, and BM lesions were all located in the brain parenchyma; ④ The diameter of tumors was >5 mm (20); ⑤ T2WI and CE-T1 examinations were performed on preoperative MRI. Exclusion criteria: ① pathologically confirmed other types of tumors; ② no MRI examination before surgery; ③ unclear tumor boundaries and unable to accurately delineate the volume of interest (VOI); ④ patients had received radiotherapy, chemotherapy or hormone therapy before surgery; ⑤ had a history of other brain tumors or trauma. ⑥ patients and family members disagreed. See **Figure 1** for the flow chart.

### 2.3 MRI data

All patients included in the study underwent MRI image acquisition before surgery using Siemens MRI equipment, including 1.5T magnetic field strength with 8-channel head phased array coil and 3.0T magnetic field strength with 16-channel head phased array coil. The following are the detailed scanning parameters and contrast agent usage: CE-T1 scanning parameters: TR/TE are 1600 ms/3.02 ms and 1700 ms/24 ms, FOV are 210 mm×210 mm and 240 mm×240 mm, slice thickness are 5.0 mm and 6.0 mm, and slice spacing is 5.5 mm and 6.5 mm respectively; T2 scanning parameters: TR/TE are 5400 ms/98 ms and 5700ms/93 ms, FOV are 210 mm×210 mm and 240 mm×240 mm, slice thickness are 5.0 mm and 6.0 mm, and slice spacing is 5.5 mm and 7.5 mm respectively. These image data were then used for subsequent radiomics feature extraction and development of machine learning models.

### 2.4 MRI data processing and feature extraction

#### 2.4.1 Image preprocessing

We used Python (3.7. 1) to apply the N4ITK bias correction algorithm to all images to avoid uneven signal intensity. The images were then resampled to a standardized voxel spacing of 1 mm × 1 mm × 1 mm, and the voxel intensity was discretized (25 Hu bin) to reduce image noise and standardize intensity. Finally, the images were normalized to the maximum and minimum values of signal intensity to reduce the difference in signal intensity between images acquired by different machines.

#### 2.4.2 Image segmentation and feature extraction

We registered T2WI to CE-T1 based on the General Registration (Elastix) module of 3D Slicer (http://www.Slicer.org, version 5.7.0) software. A primary neurosurgeon and a primary radiologist jointly agreed to outline the VOI layer by layer on the registered CE-T1 image combined with the original image. The VOI includes all information such as the tumor core, enhancement area, necrosis and cystic changes. The tumor VOI outlined based on the CE-T1WI image was copied and registered with the brain tumor on the T2WI image to obtain the brain tumor VOI based on the T2WI image. Then, we used the Radiomics module of 3D Slicer to extract features from the VOI of each image, including first-order features, morphological features, texture features (grayscale co-occurrence matrix, grayscale region size matrix, grayscale dependency matrix, neighborhood grayscale difference matrix, grayscale run-length matrix) and wavelet transform. A total of 851 radiomics features were extracted for each patient.

#### 2.4.3 Data preprocessing and feature screening

We performed Z-score standardization on the features extracted from the images to ensure the consistency and comparability of the data among different features, laying the foundation for subsequent analysis work. To verify the consistency of the texture feature extraction process, we reviewed the results evaluated using the intraclass correlation coefficient (ICC) and selected features with ICC values >0.75, which were considered to be highly relevant when independently extracted by two doctors. consistency and reliability, and therefore were used in subsequent analysis and model building. Next, we used the F test to conduct a retrospective analysis of the historical data, with the purpose of eliminating those features that were not significantly different between different imaging groups (i.e., P value > 0.01), ensuring that only those features that were statistically significant in distinguishing different imaging groups were characteristics are retained. Subsequently, we used recursive feature elimination (RFE) to reduce the dimensionality of the remaining features (the target dimension was set to 10). Finally, we built a model based on these rigorously screened and dimensionally reduced features. This model has not only demonstrated high prediction accuracy and robustness in the past, but its construction process has also fully demonstrated our rigor and scientificity in feature selection, ensuring the validity and reliability of the model.

#### 2.4.4 Establishment and verification of ML models

**Figure 2:**
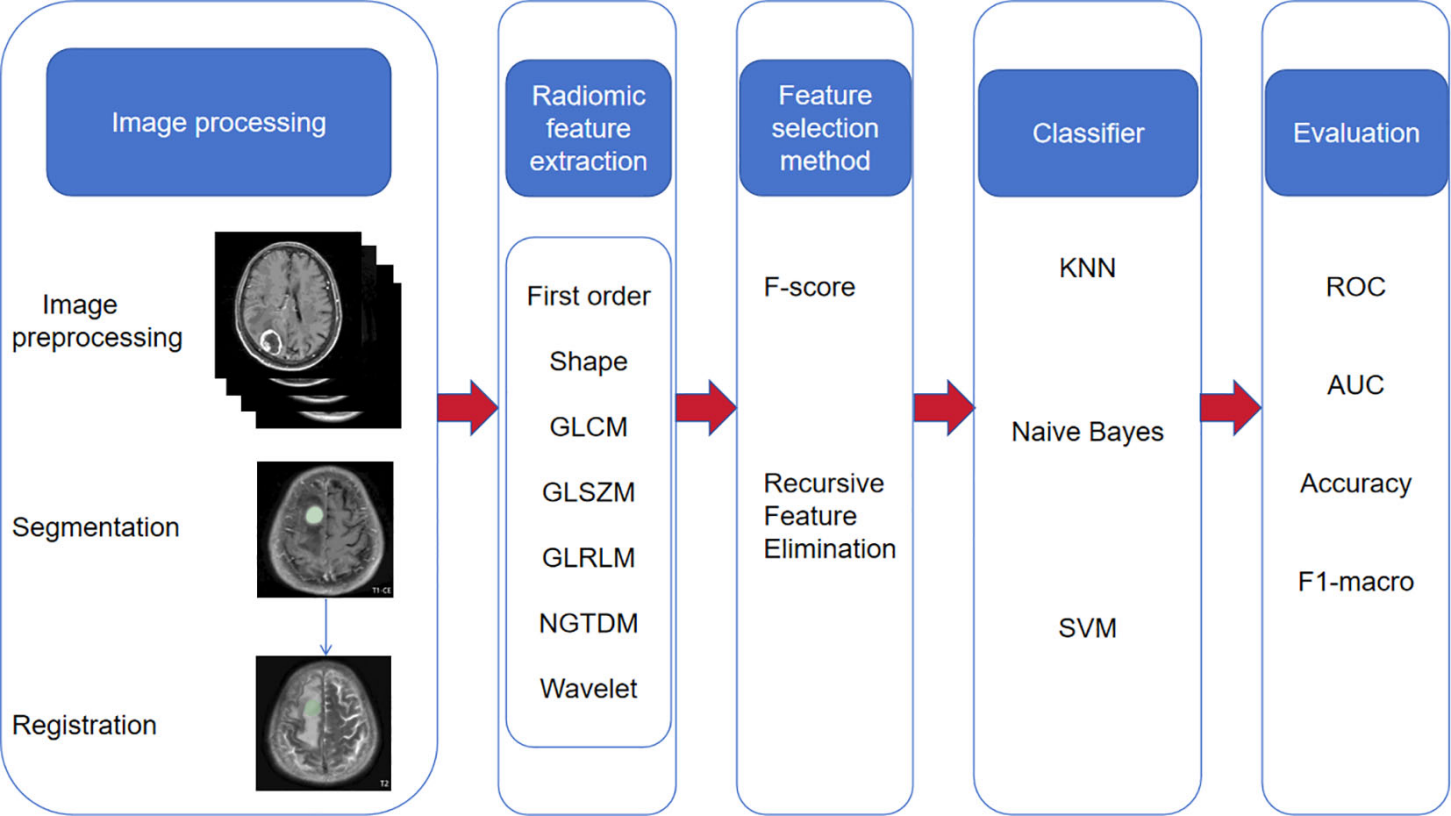
Research route.

We randomly divided the case data into a training set (containing 141 samples) and a test set (containing 61 samples) in a ratio of 7:3. Subsequently, we adopted the One-Vs-Rest (OvR) multi-classification strategy and trained three machine learning models using the training set data: support vector machine (SVM), K nearest neighbor classification (KNN), and Naive Bayes classifier(NBC). In order to optimize the performance of the model, we used a 5-fold cross-validation technique to select the best hyperparameter combination on the training set. During the model construction process, we considered models based on a single MRI sequence (such as T2 sequence or CE-T1 sequence), and also explored the joint model of T2 sequence combined with CE-T1 sequence to evaluate the impact of different input data on model performance. After training, we used the validation set to evaluate the performance of the three machine learning models. The entire process, including data partitioning, model training, hyperparameter optimization, model evaluation and other steps, was implemented using the Python programming language. The research roadmap is shown in **Figure 2**.

### 2.5 Statistical methods

For measurement data (i.e., continuous variables or numerical data), we use mean (Mean) **±** standard deviation (Standard Deviation, SD), i.e. (X **±** S) for description. In the data analysis stage, if the data does not meet the assumption of normal distribution or homogeneity of variance, we may choose Kruskal-Wallis H test (when comparing three or more independent samples) for non-parametric test. For count data (i.e., categorical variables or discrete data), we use frequency (n) and percentage (%) to express its distribution. When analyzing such data, Chi-square test is a commonly used method to compare whether the differences between different classification groups are statistically significant. When constructing a multi-classification model, in order to evaluate the overall classification performance of the model, we use the classification-weighted macro-average receiver operating characteristic (ROC) curve and the area under the curve (AUC) as evaluation indicators. These indicators can comprehensively reflect the classification ability of the model in different categories. In order to compare the performance differences of different machine learning models in classification tasks, especially the differences in AUC values, we use the DeLong test. This is a statistical method specifically used to compare whether the AUC values under two or more ROC curves are significantly different. Finally, we set the significance level to 0.05, that is, when the P value is less than 0.05, we believe that the observed difference is statistically significant.

## 3 Results

### 3.1 Patient characteristics

A total of 202 patients were included in the study, comprising 71 patients (35.1%) diagnosed with WHO grade 4 GB (IDH-wildtype), including 36 women (50.7%) and 35 men (49.3%), with a mean age of 60.1 ± 15.5 years, ranging from 30 to 80 years. Additionally, 59 patients (29.2%) had PCNSL, all of which were diffuse large B-cell lymphoma (DLBCL-PCNSL), including 29 women (49.2%) and 30 men (50.8%), with a mean age of 61.0 ± 14.6 years, ranging from 30 to 85 years. Furthermore, 72 patients (35.6%) had brain metastases originating from various primary tumors, including 34 patients with lung cancer (47.2%), 12 patients with breast cancer (16.7%), 3 patients with melanoma (4.2%), 14 patients with digestive tract tumors (19.4%), 8 patients with renal cancer (11.1%), and 1 patient with fibrosarcoma (1.4%). The mean age of these patients was 62.4 ± 14.6 years, ranging from 35 to 85 years. These patients were selected as the training set and validation set for the ML classifier. The overall patient demographics are shown in **Table 1**.

**Table 1:** Demographics and clinical characteristics of the patients.

	Training set				Test set			
	GBM	PCNSL	BM	P value	GBM	PCNSL	BM	P value
Patients	51	39	51		20	20	21	
Age	54.4 ± 15.2	62.8 ± 15.0	62.3 ± 15.9	0.13	56.6 ± 13.0	56.3 ± 14.0	61.6 ± 13.6	0.33
Female	23	17	27	0.618	13	12	12	0.874

### 3.2 Selected radiomic features

We used the Radiomics module of 3D Slicer to extract 851 radiomic features from the CE-T1 and T2 sequences for each patient, including first-order features, morphological features, texture features, and wavelet features. After filtering the radiomic features through intragroup correlation (ICC>0.75), F test (P<0.01), and recursive feature elimination (target dimension set to 10), the feature sets of CE-T1, T2, and CE-T1 combined with T2 each contained 10 features. Among the features extracted from the joint parameters, a total of 7 CE-T1 features and 3 T2 features were selected, as shown in **Table 2**.

**Table 2:** Features retained after screening by CE-T1, T2, and CE-T1 combined with T2 models.

T2	CE-T1	CE-T1 combine T2
T2-Original-shape-Sphericity	T1-wavelet-LHL-gldm-LargeDependenceLowGrayLevelEmphasis	T1-wavelet-LHL-gldm-LargeDependenceLowGrayLevelEmphasis
T2-Original-shape-Maximum2DDiameterSlice	T1-original-firstorder-Skewness	T1-original-firstorder-Skewness
T2-wavelet-LHL-glrlmRunVariance	T1-original-shape-Flatness	T1-original-shape-Sphericity
T2-wavelet-LHL-glrlm-LongRunLowGrayLevelEmphasis	T1-original-shape-Sphericity	T1-wavelet-LLH-gldm-LargeDependenceLowGrayLevelEmphasis
T2-wavelet-LHL-gldm-LargeDependenceHighGrayLevelEmphasis	T1-wavelet-LLH-gldm-LargeDependenceLowGrayLevelEmphasis	T1-wavelet-LHL-glcm-JointAverage
T2-wavelet-HLL-gldm-LargeDependenceHighGrayLevelEmphasis	T1-wavelet-LHL-gldm-LowGrayLevelEmphasi	T1-wavelet-LLL-firstorder-Skewness
T2-wavelet-LLH-ngtdmContrast	T1-wavelet-LL-LfirstorderSkewness	T1-wavelet-HLL-gldm-DependenceNonUniformityNormalized
T2-wavelet-HHL-glcmMCC_	T1-wavelet-HLL-gldm-DependenceNonUniformityNormalized	T2-Original-shape-Sphericity
T2-wavelet-LLH-firstorderKurtosis	T1-wavelet-LLH-glszm-GrayLevelNonUniformity	T2-wavelet-HLL-gldm-LargeDependenceHighGrayLevelEmphasis
T2-wavelet-HLL-glrlmShortRunHighGrayLevelEmphasis	T1-wavelet-LLL-firstorder-Minimum	T2-wavelet-LLH-ngtdm-Complexity

### 3.3 Machine learning model effectiveness evaluation

#### 3.3.1 Performance of a single sequence model

CE-T1 sequence: When comparing the performance of three classifiers (SVM, KNN, and NBC), we found that the SVM model performed best on a number of key indicators. Specifically, the macro-average AUC of the SVM model reached 0.91, which is significantly higher than the 0.86 of KNN and the 0.82 of NBC, showing the advantages of SVM in handling complex classification tasks. In addition, the accuracy of the SVM model reached 72. 1%, which is also the highest among the three classifiers. The F1 score, as the harmonic mean of accuracy and recall, is also an important indicator of classifier performance. The F1 score of SVM is 0.719, which is also ahead of KNN and NBC. Although KNN and NBC also show certain classification capabilities, their performance is slightly inferior to SVM. This may be due to the stronger generalization ability of SVM when dealing with high-dimensional data and complex classification boundaries, while KNN may be affected by noisy data and the curse of dimensionality, and NBC assumes the independence between features, This is often not true in actual situations. In summary, the SVM model stands out among the three classifiers, and its excellent performance provides strong support for subsequent clinical applications and research.

**Figure 3:**
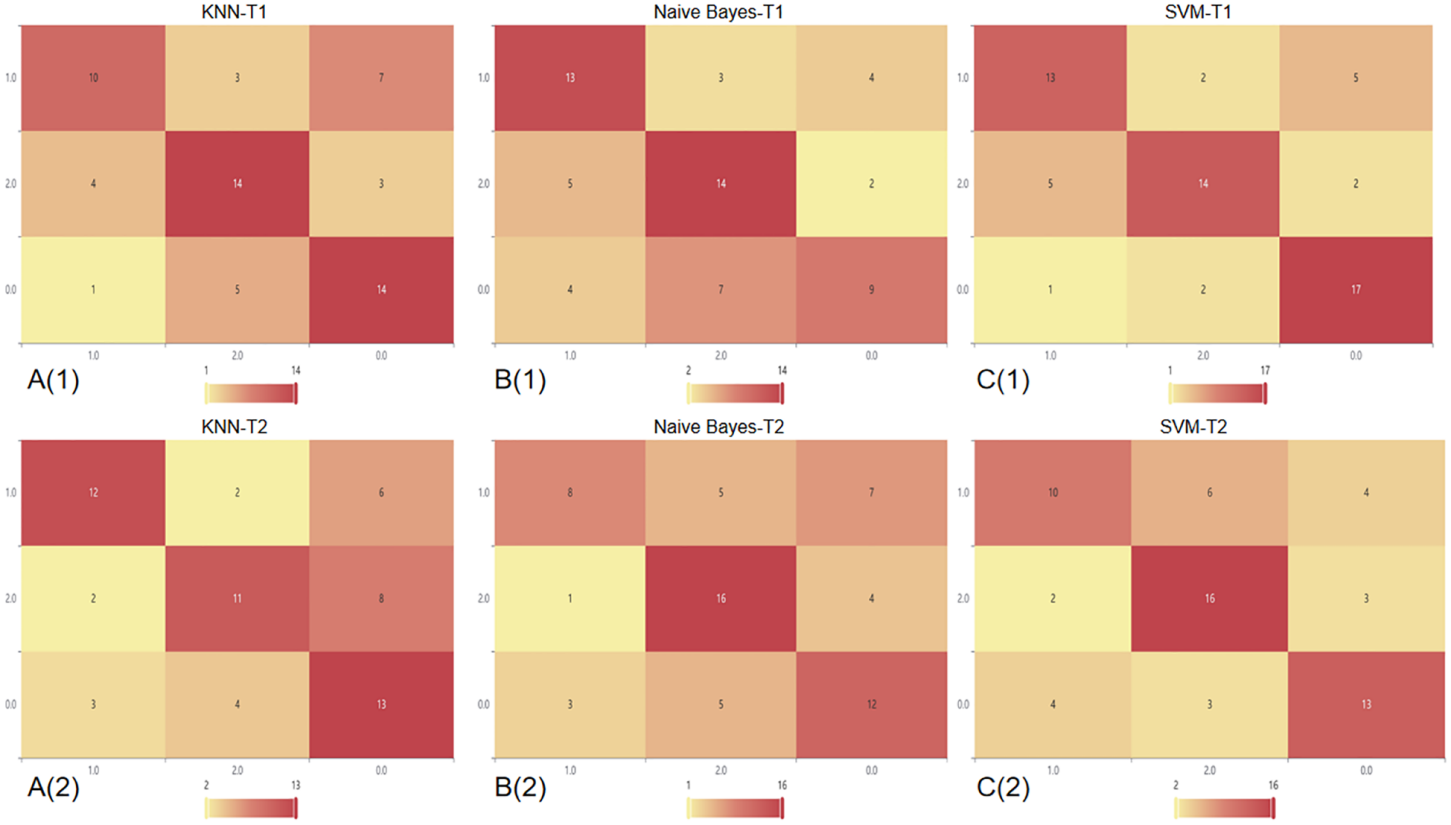
Confusion matrix heat map of a single sequence in three classifier models. In this context, the number 0 represents BM, 1 represents GB, and 2 represents PCNSL. **A(1)**: Confusion matrix of CE-T1 in the KNN; **A(2)**: Confusion matrix of T2 sequence in the KNN; **B(1)**: Confusion matrix of CE-T1 in the NBC; **B(2)**: Confusion matrix of T2 sequence in the NBC; **C(1)**: Confusion matrix of CE-T1 in the SVM; **C(2)**: Confusion matrix of T2 sequence in the SVM.

**Figure 4:**
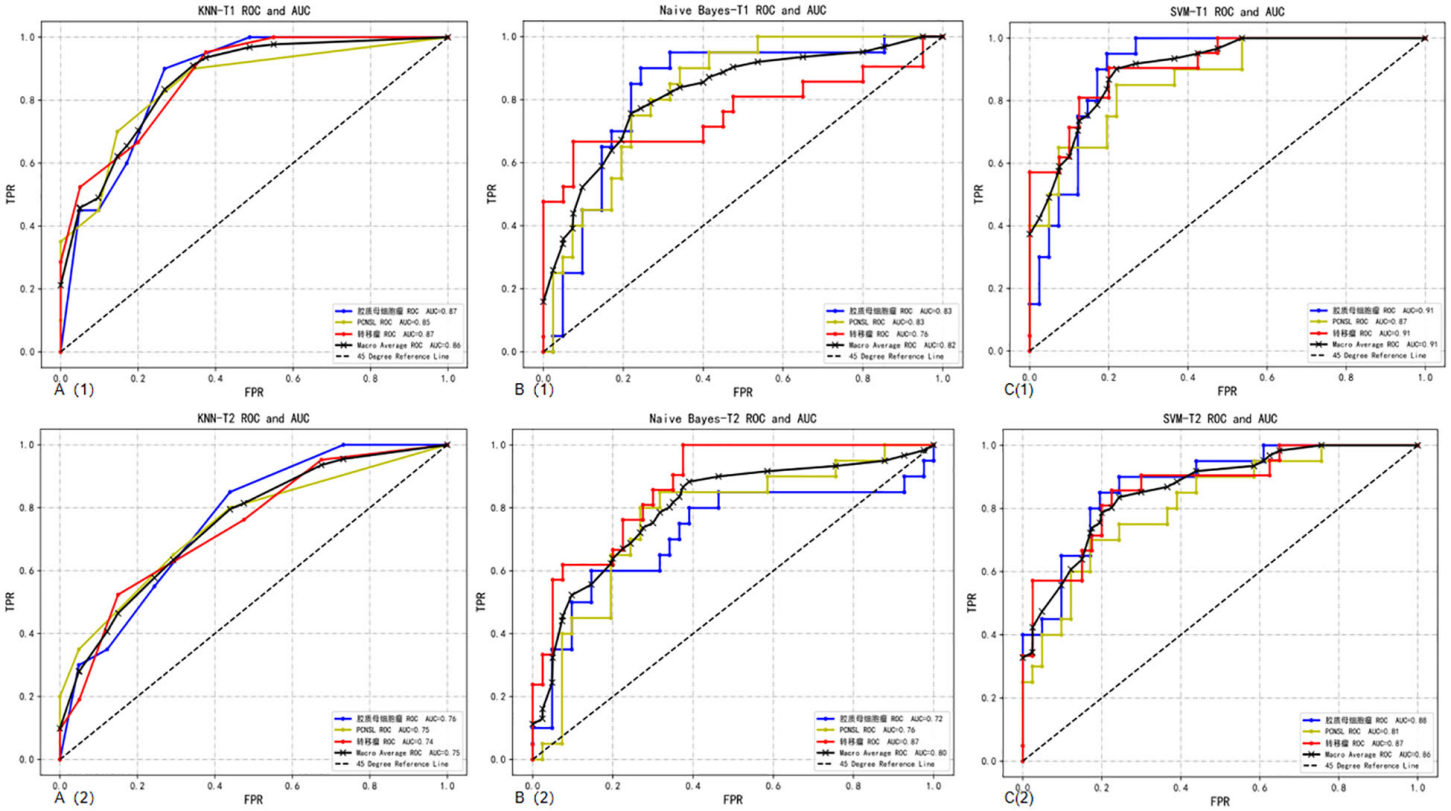
Macro-average ROC curves and AUCs of a single sequence in three classifier models. **A(1)**: ROC curve of CE-T1 sequence in the KNN; **A(2)**: ROC curve of T2 sequence in the KNN; **B(1)**: ROC curve of CE-T1 sequence in the NBC; **B(2)**: ROC curve of T2 sequence in the NBC; **C(1)**: ROC curve of CE-T1 sequence in the SVM; **C(2)**: ROC curve of T2 sequence in the SVM.

T2 sequence: When comparing the performance of models built based on the T2 sequence and the CE-T1 sequence, we found that when the T2 sequence was used alone, the performance of the constructed model was slightly lower than that of the CE-T1 sequence. Specifically, the AUC of the SVM model trained using the T2 sequence was 0.86, which is still a relatively high value, but lower than the performance of the SVM model under the CE-T1 sequence. Similarly, the KNN and NBC models also achieved AUCs of 0.75 and 0.80, respectively, when using the T2 sequence, which are also lower than their performance under the CE-T1 sequence. To more intuitively demonstrate these differences, we provide the confusion matrix heat map and macro-average ROC curve of each model constructed under the T2 sequence (as shown in **Figures 3**, **4**). The confusion matrix heat map reflects the prediction accuracy of the model in each category through the depth of color, while the ROC curve shows the true positive rate (TPR) and false positive rate (FPR) of the model at different thresholds. The AUC value quantifies the area under the ROC curve and is used to evaluate the overall performance of the model. These results suggest that although the T2 sequence also contains certain diagnostic information, the CE-T1 sequence may contain more features that contribute to the classification task, or these features are more significantly and stably expressed in the CE-T1 sequence. Therefore, in practical applications, if conditions permit, we can consider combining the features of multiple MRI sequences to build a more accurate and robust classification model.

#### 3.3.2 Performance of combined sequences model

**Figure 5:**
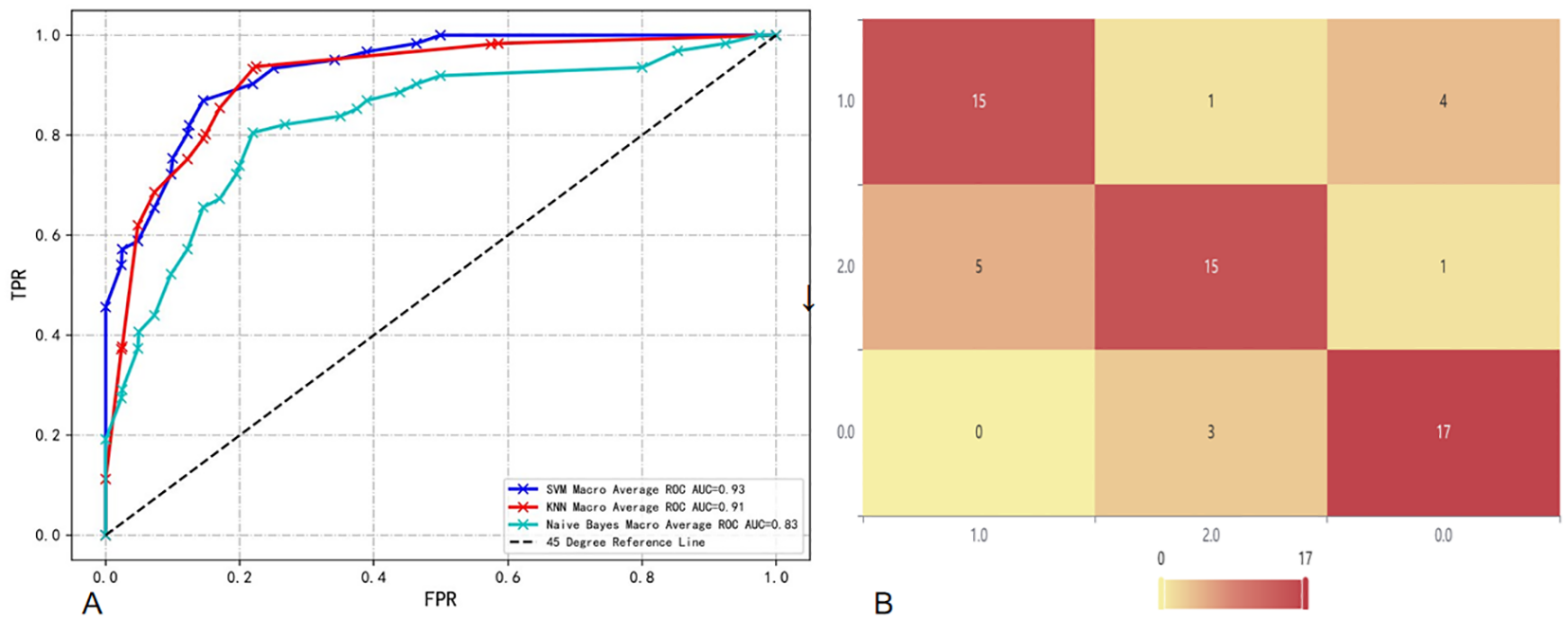
**(A)** Macro-average ROC and AUC of combined sequences in different classifier models; **(B)** Confusion matrix heat map of combined sequences in SVM.

T2 combined CE-T1 sequence: When we combine the features of T2 and CE-T1 MRI sequences, the macro-average AUC of the SVM model significantly increases to 0.93. This result clearly demonstrates the advantages of the combined sequence in improving diagnostic performance. Furthermore, the accuracy of the joint model reached 77%, and the F1 score was also 0.77 (**Table 3**). These indicators jointly prove the effectiveness of the joint sequence in the classification task. Specific to the classification performance of the SVM model, for the classification of GB, its sensitivity reached 85% and specificity was 87.8%, indicating that the model has high accuracy and stability in identifying GB. At the same time, the sensitivity for DLBCL-PCNSL is 75%, and the specificity remains at 87.8%, showing that the model has good discrimination ability for different types of central nervous system tumors. The sensitivity for BM is 71.4%, and the specificity is further improved to 90%. Although the sensitivity is slightly lower than the other two types of lesions, the high specificity indicates that the model is very accurate in confirming non-BM cases. In order to more intuitively demonstrate the performance of joint parameters in the SVM model, we provide a heat map of the confusion matrix, which intuitively reflects the consistency between the model’s predictions and actual results on each category through the depth of the color. In addition, we also plotted the macro-average ROC curve (shown in **Figure 5**) and the area under the ROC curve (AUC) of the joint parameters in the three classifier models. Among them, the SVM model had the highest AUC value (0.93). The significant role of joint sequences in improving the overall performance of the model is further verified. Together, these results support the effectiveness of combining T2 and CE-T1 sequence features in building high-performance central nervous system tumor classification models.

**Table 3:** Evaluation results of the SVM using the combined CE-T1 and T2 sequences.

	Accuracy	Recall	Precision	F1 Score
Training set	0.901	0.901	0.901	0.901
Test set	0.770	0.770	0.771	0.770

#### 3.3.3 Model comparison and analysis

The results of the DeLong test show that when we use the features of T2 and CE-T1 sequences together, the model constructed has a significantly higher AUC value than the model using only a single sequence (T2 or CE-T1), and this The difference is statistically significant (P<0.05). In further analysis, we found that the SVM performed particularly well when utilizing joint sequence models. Not only has its AUC value been significantly improved, but the SVM model has also achieved significant enhancements in the classification performance of GB, DLBCL-PCNSL and BM. This shows that the SVM model can more effectively extract and utilize complementary information from combined sequences, thereby improving the recognition accuracy of different types of brain lesions. Finally, the evaluation results on the test set showed that the SVM showed the best prediction ability when using combined sequences for prediction. This finding not only verifies the effectiveness of the combined sequence model, but also emphasizes the superiority of the SVM classifier in handling such complex classification tasks.

## 4 Discussion

Based on preoperative multi-parametric MRI radiomics, we developed and verified a non-invasive auxiliary method to identify three common central nervous system tumors GB by extracting features of CE-T1 and T2 MRI sequences and combining it with ML methods., DLBCL-PCNSL and BM models, mainly include SVM model, KNN and NBC. Among them, the SVM not only shows the highest AUC in the three-classification task value (0.93), and its accuracy, sensitivity and specificity are significantly better than the KNN and NBC, and it has significant advantages in diagnostic performance. The research results further confirm the importance of combining MRI information fusion with advanced machine learning algorithms (21), providing objective clinical evidence-based medical evidence for the accurate identification of complex central nervous system tumor types and the formulation of diagnosis and treatment plans.

In previous studies, P Alcaide-Leon et al. (22) extracted CE-T1 sequences from 71 GB and 35 DLBCL-PCNSL patients to establish an SVM. The results showed that the SVM based on CE-T1WI texture features was not inferior to expert evaluation in distinguishing DLBCL-PCNSL from GB. Zenghui Qian et al. (23) used different machine learning models to identify 242 GB and 170 BM patients and found that the SVM + least absolute shrinkage and selection operator (LASSO) classifier had the highest predictive effect with an AUC of 0.9. In addition, its clinical performance was superior to that of neuroradiologists in terms of accuracy, sensitivity, and specificity. Swinburne et al. (24) performed multi-class classification on diffusion and perfusion MR images and conventional MR images of 26 patients with GB, BM or PCNSL, and evaluated support vector classifiers and multi-layer perceptron models, with the highest accuracy of 69.2%. However, these models did not use radiological features, and the validation method did not use an independent validation set. Bio Joo et al. (25) established a machine learning model based on CE-T1 combined with T2 for multi-class classification of GB, PSNCL and BM. The macro-average AUC of the best model was 0.878, but the study did not extract wavelet during feature extraction. This study found that the model established after extracting wavelet features improved the diagnostic efficacy of the model. Wavelet can filter and denoise the original image, so these transformed features can effectively capture key tumor heterogeneity and better predict tumor biology. Wavelet may reflect certain cytological characteristics of the tumor microenvironment or the specific expression of certain molecules (26).

**Figure 6:**
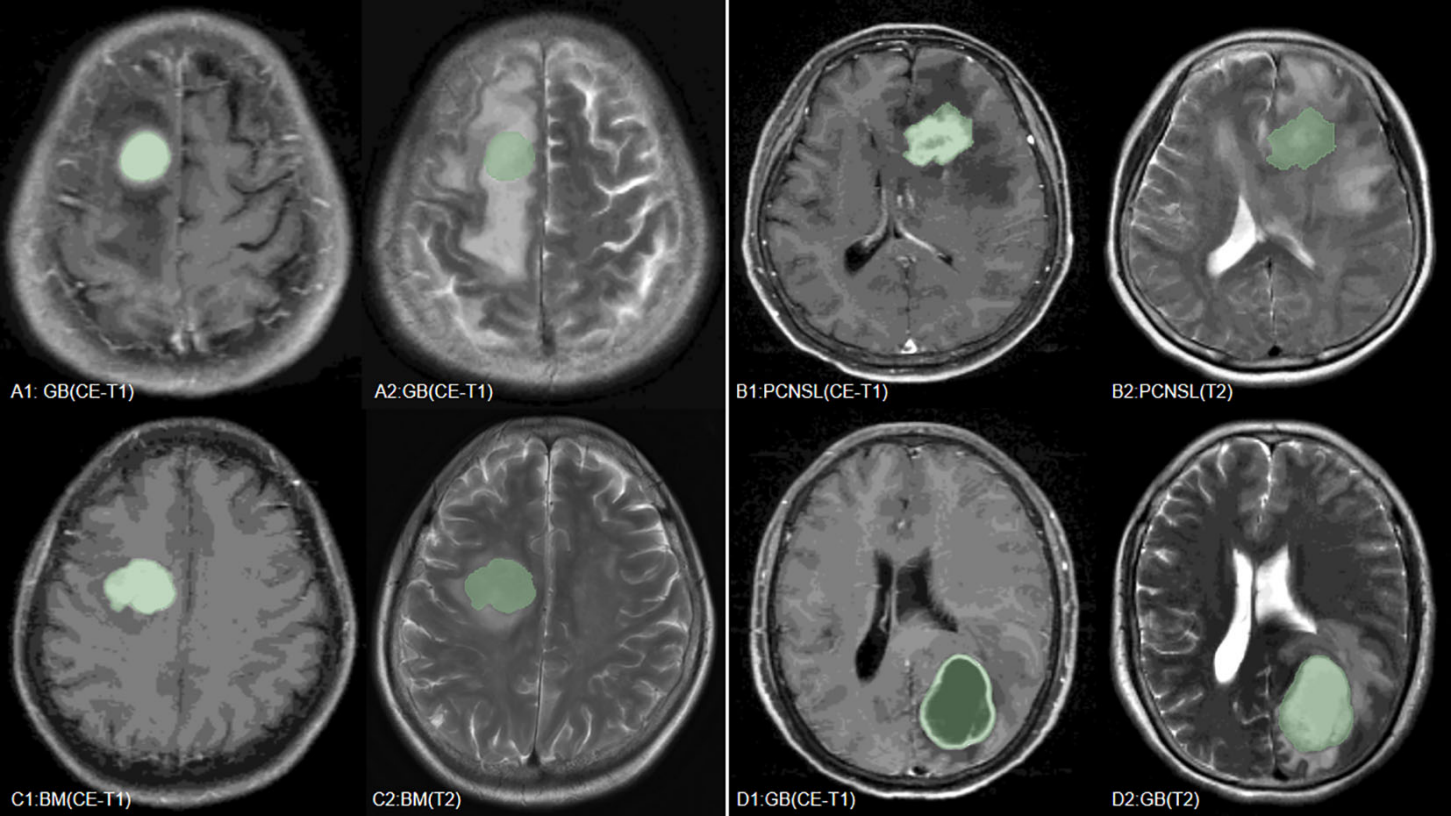
We have listed MRI images of four cases, where **(A–C)** are the GB, PCNSL, and BM correctly predicted by the model, respectively, and **(D)** is a case where the model incorrectly predicted BM as GB.

Our study found that the combined use of CE-T1 and T2 MRI sequences has significant advantages in improving the classification and identification performance of CNS tumors. In the multi-classification task of the combined model, whether it is sensitivity, specificity or AUC value, it is significantly better than the model based on a single sequence, which fully proves that the combination of multi-classification technology can improve the diagnostic performance of the model. potential. This conclusion has been fully verified on multiple evaluation indicators such as the AUC value and accuracy of the model (27). It is particularly worth noting that the SVM model shows the best diagnostic performance when processing joint sequence data, which is similar to previous research results (18). Through in-depth research, we found that wavelet-LHLgldmLargeDependenceLowGrayLevelEmphasis stands out as a key feature in tumor classification. Specifically, the value of this feature is significantly higher in GB compared to BM and PCNSL, which may be related to the larger necrotic areas present in GB, while the necrotic areas in BM and PCNSL are relatively smaller (28). However, since some brain metastases also have larger necrotic areas (29), the value of wavelet-LHLgldmLargeDependenceLowGrayLevelEmphasis in some brain metastases is also higher, which may be part of the reason for model classification errors (**Table 4**, **Figure 6**). In addition, this study also successfully introduced multi-classification technology into the differential diagnosis of central nervous system tumors, allowing the model to handle three different types of tumors at the same time, and is no longer limited to previous two-classification tasks. By adopting the OneVsRest multi-classification strategy, the model can make full use of various image features (such as GLCM, GLSZM, Wavelet features, etc.) to classify tumor types more accurately and carefully. In addition, the number and types of features extracted from the combined sequences are richer, allowing the model to evaluate tumor heterogeneity from multiple dimensions, further enhancing the robustness and clinical application potential of the model, allowing the model to be combined with multiple parameters Effectively handle more complex tasks, showing higher generalization ability and diagnostic efficiency.

**Table 4:** We have listed three correctly classified cases and one incorrectly classified case, along with the corresponding features.

Prediction results	PCNSL	BM	GB	GB
Actual results	PCNSL	BM	GB	BM
T1wavelet-LHLgldmLargeDependenceLowGrayLevelEmphasis	-0.783045612	-0.434221917	1.660564694	0.033418416
T1originalfirstorderSkewness	1.716216714	0.343341327	-0.648128803	-0.247728039
T1originalshapeSphericity	1.188091697	-0.368825139	-2.086246787	-1.23875299
T1wavelet-LLHgldmLargeDependenceLowGrayLevelEmphasis	0.65578243	-0.664195776	-0.161672473	0.041384592
T1wavelet-LHLglcmJointAverage	0.708196618	0.739044205	-1.841120733	-0.419397948
T1wavelet-LLLfirstorderSkewness	1.512422687	0.274490834	-0.552201118	-0.218504389
T1wavelet-HLLgldmDependenceNonUniformityNormalized	0.587596769	0.029673098	-2.225515335	-0.043338455
originalshapeSphericity	1.187273887	-0.437288232	-2.190445966	-1.315302084
wavelet-HLLgldmLargeDependenceHighGrayLevelEmphasis	0.482900455	0.273711743	-0.400932721	-0.540081324
wavelet-LLHngtdmComplexity	0.530989434	-0.603025579	1.043153293	0.327984142

Their MRI images are shown in **Figure 6**.

Although this study has achieved encouraging results, it still has some limitations. First, this is a single-center retrospective study and lacks multicenter data verification. More external data should be introduced in the future to verify the universality of the model. In addition, the sample size is relatively small, and the sample size can be further expanded in the future to improve the robustness and generalization ability of the model. And, this study only used CE-T1 and T2 sequences, and did not combine other sequences (such as DSC, PWI, etc.). Recent studies have proposed a new voxel-wise classification method based on DSC perfusion data (30). Future studies can try to incorporate more sequences into the model to further improve its diagnostic efficacy. And although the model demonstrated good diagnostic performance on the validation set, it indeed lacks an in-depth explanation of the pathological basis of key features. In future studies, we plan to incorporate histopathological data by integrating pathological slides and molecular markers to explore the direct associations between imaging features and pathological characteristics.

## 5 Conclusion

This study successfully developed and validated three machine learning models based on preoperative MRI radiomics features, which showed good performance in distinguishing GB, DLBCL-PCNSL and BM. The model mainly includes SVM, KNN and NBC. Among them, the SVM not only shows the highest AUC value (0.93) in the three-classification task, Moreover, its accuracy, sensitivity and specificity are significantly better than KNN and NBC, and it has significant advantages in diagnostic performance. It not only performs well in the classification task of a single sequence (CE-T1, T2), but also achieves the best performance in the classification performance of combined sequences. In addition to this, the combined sequences model improves the diagnostic performance compared to the single sequence model. With the continuous development of imaging technology and the continuous optimization of machine learning algorithms, this kind of machine learning model based on MRI radiomics features will play a more important role in the diagnosis and treatment of central nervous system tumors.

## Data Availability

The raw data supporting the conclusions of this article will be made available by the authors, without undue reservation.
